# Effect of dietary inclusion of gamma-ray irradiated black soldier fly larvae or grasshopper on blood metabolites and immunity in broiler chickens

**DOI:** 10.1016/j.psj.2025.106012

**Published:** 2025-10-24

**Authors:** Ali Reyan Mohassesi, Hassan Darmani Kuhi, Ardeshir Mohit, Shahrokh Ghovvati

**Affiliations:** Department of Animal Science, Faculty of Agricultural Sciences, University of Guilan, Rasht, Iran

**Keywords:** Black soldier fly larvae, Grasshopper, Immune response, Broiler chickens, Gamma-irradiated

## Abstract

This study aimed to evaluate the effects of replacing soybean meal with Black Soldier Fly Larvae (BSFL)/Grasshopper (GH) powders on health, mortality rate, blood metabolites, immune responses, and antioxidant enzyme activities in broiler chickens. A total of 546 male broiler chicks were randomly assigned to seven dietary treatments in a completely randomized design (*n* = 78 birds per treatment with 6 floor-pen replicates of 13 birds each). The control group was fed a conventional diet based on corn and soybean meal. In the test groups, soybean meal was progressively substituted with BSFL/GH powders at the levels of 8, 16, and 24 %. All insect meals used in the study were gamma-irradiated prior to inclusion The economic efficiency of the different dietary treatments was assessed using the European Production Efficiency Factor (EPEF). Broilers fed 24 % BSFL exhibited the highest EPEF across all growth phases (*p* < 0.05), with notable improvements also observed in birds receiving 16 % BSFL and 16–24 % GH. Mortality rates were lower in insect-fed groups than the control group. Diets containing BSFL significantly lowered serum cholesterol, triglycerides, and very low-density lipoprotein (VLDL) levels (*p* < 0.05). Immune responses, including antibody titers against Avian Influenza (AI), Newcastle Disease (ND), and Sheep Red Blood Cells (SRBC) on days 28 and 42, were significantly enhanced in birds receiving 24 % BSFL. White blood cell counts were not affected by dietary treatment (*p* > 0.05). Activities of antioxidant enzymes **-**glutathione peroxidase (GSH-Px), catalase (CAT), and superoxide dismutase (SOD)**-** and oxidative stress indicators including thiobarbituric acid reactive substances (TBARS) and total antioxidant capacity (TAC), remained unchanged (*P* > 0.05). These findings suggest that BSFL, and to a lesser extent GH, are promising, sustainable alternatives to soybean meal, enhancing broiler performance and immunity without compromising oxidative status. Gamma irradiation of insect meals may have contributed to these improvements. All insect meals were gamma-irradiated prior to inclusion; proximate composition and microbiological analyses indicated that irradiation did not compromise the nutritional quality of the insect meals. Further work should evaluate the long-term effects and commercial applicability of irradiated insect meals under commercial production conditions.

## Introduction

With the global population projected to exceed 10 billion by 2050, the demand for animal-derived proteins, especially poultry meat, is expected to rise substantially. Poultry is favored due to its affordability, efficient feed conversion, and widespread consumer acceptance (Rumpold and Schlüter, 2013). However, traditional protein sources like soybean meal(SBM) and fishmeal face increasing sustainability challenges related to environmental degradation, high production costs, and competition with human food systems (Sajid et al., 2023). In this context, insects have gained attention as a viable alternative protein source for poultry. They provide high-quality protein, essential amino acids, lipids, vitamins, and minerals, and can be produced using organic waste with a low environmental footprint (van Huis and Oonincx, 2017; Miglietta et al., 2015). Compared to conventional livestock feed, insect farming requires less land and water, emits fewer greenhouse gases, and contributes to circular agriculture by converting biowaste into high-value biomass (Oonincx et al., 2010; Davis, 2023). Beyond their nutritional profile, insect-based meals offer functional properties through components such as chitin, lauric acid, and antimicrobial peptides (AMPs). Chitin, comprising 5–20 % of insect dry matter, acts as dietary fiber and modulates immune responses via pattern recognition receptors (Elieh Ali Komi et al., 2018; Islam and Yang, 2017). These bioactives have demonstrated immunostimulatory effects, enhancing cytokine expression, lysozyme activity, and gut integrity (Ibitoye et al., 2023; Park et al., 2023).

Immune health is a critical determinant of poultry performance, particularly under conditions of stress or pathogen exposure (Alispahic et al., 2024). The use of insect-derived bioactive compounds provides a natural strategy to boost host immunity while reducing reliance on antibiotic growth promoters (Koutsos et al., 2022; Elnesr et al., 2022). This approach aligns with global efforts to mitigate antimicrobial resistance through nutritional modulation of the immune system.

Among edible insects, Black Soldier Fly Larvae (BSFL) are widely studied due to their high protein content (40–60 % on a dry matter basis), favorable amino acid profile, and functional lipids such as lauric acid (Spranghers et al., 2018; Makkar et al., 2014). Studies have shown that BSFL inclusion improves growth performance, immune responses, gut morphology, and resistance to *Salmonella Gallinarum* infection (Lee et al., 2018a; Schiavone et al., 2019). BSFL also enhance nutrient digestibility without negatively affecting mortality (Nahrowi et al., 2024; Bhattacharyya et al., 2024).

Grasshoppers (GH), belonging to the Orthoptera order, are another promising insect protein source. They are rich in protein and contain AMPs with antimicrobial properties (Elahi et al., 2022; Chae et al., 2012). Though less researched than BSFL, GH have shown potential to improve feed efficiency, growth, and immune modulation, especially in arid and resource-limited environments (Lee et al., 2024).

To ensure microbial safety and improve nutrient utilization of insect meals, gamma irradiation has been proposed as an effective post-harvest treatment. It eliminates microbial contaminants such as *Aspergillus* and *Penicillium* at doses of 3–7 kGy (Byun et al., 1988), extending shelf life and maintaining nutritional quality without chemical preservatives (Kierończyk et al., 2018). Additionally, gamma irradiation enhances fat digestibility, reduces oxidation, and may increase nutrient bioavailability in insect-based feeds, making it especially relevant for feed safety and quality control. Recent advances also include selective breeding and genomic tools to optimize the nutritional composition and functional properties of insects for feed applications (Eriksson and Picard, 2021). These innovations, alongside regulatory shifts such as the EU’s approval of insect meals for aquaculture and poultry, support the integration of insects into mainstream livestock nutrition (Committee, 2015).

Despite growing interest, further research is needed to understand how insect meals affect specific immunological and metabolic markers in poultry, particularly in antibiotic-free systems. Key indicators such as antibody titers (e.g., SRBC), antioxidant capacity, and blood metabolites remain underexplored in the context of irradiated insect meals.

Therefore, the objective of this study was to investigate the effects of dietary inclusion of gamma-irradiated BSFL and GH on growth performance, blood metabolites, immune responses, and oxidative status in broiler chickens. The study aimed to evaluate the nutritional and functional efficacy of these novel protein sources and to assess gamma irradiation as a tool to enhance their safety and biofunctional impact.

## Materials and methods

### Experimental diets

#### Insect powder preparation

BSFL and GH were obtained from certified suppliers that rear and breed these insects in hygienic facilities under controlled conditions. Both BSFL and GH were bred up to the F14 generation to enhance production efficiency, nutritive quality, and consistency as feed ingredients.

#### Gamma irradiation

Gamma irradiation of BSFL and GH powders was carried out at a licensed facility using a Cobalt-60 (⁶⁰Co) source. Samples were exposed to three irradiation doses: 0 kGy (control), 5 kGy, and 10 kGy. For each dose, the powders were sealed in sterile polyethylene bags and irradiated at room temperature. Dosimeters were used to confirm the accuracy of the delivered doses. To ensure uniform exposure, samples were rotated halfway through the irradiation process. After treatment, they were stabilized at room temperature for 24 h to dissipate any residual heat and radiolytic byproducts. The irradiated samples were then tested for sterility and stored at 20 °C until use in broiler diets. Microbial tests were performed to evaluate total bacterial, mold, and yeast counts. Additionally, feed composition was analyzed to detect any potential changes in nutritional value resulting from gamma irradiation (Table 1).

**Table 1:** Proximate analysis of soybean meal (SBM), non-irradiated (0 kGy) and gamma-irradiated (10 kGy) Black Soldier Fly Larvae (BSFL) and Grasshopper (GH) powders.

	SBM	BSFL 0 kGy	BSFL 5 kGy	BSFL 10 kGy	GH 0 kGy	GH 5 kGy	GH 10 kGy
Crude protein	40.90	60.94	61.32	61.47	58.43	58.17	58.65
Crude fiber	2.80	7.88	8.4	8.00	7.47	7.53	7.30
Dry matter	89.30	95.08	94.5	95.00	95.60	95.7	95.53
Ash	5.86	8.44	8.51	8.12	8.70	8.31	8.50
Total fat	2.30	4.81	4.58	4.70	5.09	5.34	5.20
Calcium	1.28	1.72	1.71	1.72	1.04	1.05	1.04
Phosphorus	0.67	0.83	0.84	0.83	0.79	0.78	0.78

#### Microbial analysis

The total microbial count in each sample was determined following irradiation. Serial dilutions were prepared, and aliquots were plated onto nutrient agar. The plates were incubated at 37 °C for 24–48 h, after which the colonies were counted and expressed as colony-forming units (CFU) per gram of sample.

### Birds and management

A total of 546 one-day-old male broiler chicks (Arian strain) were obtained from a commercial hatchery and randomly distributed across 42 pens (13 chicks/pen) at the experimental facility. Each pen (1.45 × 1.45 m) was equipped with one feeder (1.42 × 0.2 m), a drinking line with three nipples and cups, and a 1 cm layer of wood shavings. Feed and water were offered ad libitum throughout the 42-day trial. All birds received standard vaccinations. Temperature was gradually reduced from 34 °C on day 1 to 20 °C by day 42, with standard ventilation and humidity conditions maintained. Seven isonitrogenous and isocaloric diets were formulated to meet broiler requirements. The treatments included a basal diet (Control), and diets with 8 %, 16 %, or 24 % replacement of soybean meal with either BSFL powder (BSFL8, BSFL16, BSFL24) or GH powder (GH8, GH16, GH24) (Table 4). The inclusion levels (8 %, 16 %, and 24 %) were selected based on previous reports (typically up to 20 %) and to evaluate the upper practical threshold for irradiated insect meal incorporation in broiler diets.

### Collection of samples and measurements

#### Feed and production efficacy

Feed intake (FI) and bird’s mortality were recorded on daily base in each pen. Live body weight (BW) was measured weekly, and feed conversion ratio (FCR) and the European Production Efficiency Factor (EPEF) were calculated for the following periods: 1–14 days, 15–25 days, 26–35 days, and 36–42 days, using the formula:

$$EPEF=\frac{livability\left(\%\right)\times BW\left(kg\right)}{feed\;\;conersion\;\;ratio\;\;\times \;period\;\;duration\;\left(days\right)}\times 100$$

#### Blood anti- newcastle, influenza, and SRBC antibody titers

Blood samples were collected from the wing vein of two randomly selected birds per pen (*n* = 84) at 0, 28, and 42 days of age. The same tagged birds were sampled at all time points to allow within-bird comparison of antibody titers and to minimize individual variation Approximately 2 mL of blood was drawn into heparinized tubes at each sampling point and centrifuged at 3,000 *×* *g* for 10 min at 4 °C to separate plasma. The plasma samples were stored at −20 °C until further analysis.

##### Anti- newcastle disease virus (NDV) and influenza antibody titration

Antibody titers against NDV and influenza were determined using the hemagglutination inhibition (HI) test, following standard protocols. Serial two-fold dilutions of plasma were prepared in 96-well microtiter plates, and 25 µL of NDV or influenza antigen was added to each well. The plates were incubated at 37 °C for 30 min, and the results were recorded as the highest dilution at which hemagglutination was inhibited.

##### Sheep red blood cell (SRBC) antibody titer

The antibody response to SRBC was assessed using a direct hemagglutination assay. Birds were immunized with a 1 % SRBC suspension (1 mL/bird) via intramuscular injection at 21 days of age. Blood samples were collected on days 28 and 42 post-injection. Plasma samples were serially diluted in phosphate-buffered saline (PBS) in 96-well plates, and 25 µL of a 1 % SRBC suspension was added to each well. After incubation at room temperature for 1 h, the highest dilution showing visible agglutination was recorded as the SRBC titer.

#### Assessment of T-cell mediated immune response

The T-cell mediated immune response was evaluated on day 21 using the lymphoproliferative reaction to phytohemagglutinin P (PHA-P), following the method of Corrier and DeLoach (1990). Each bird received an intradermal injection of 0.1 mL PHA-P solution (1 mg PHA-P in 100 µL PBS) into the left wing web, while the right wing web was injected with 0.1 mL sterile PBS as a control. Wing web thickness was measured immediately before and 24 h after injection using a digital micrometer (MDC-25SX, Mitutoyo 293-821-30, Japan; accuracy 0.01 mm). The Wing Web Swelling Index (WWSI) was calculated as follows:

WWSI = (Thickness post-PHA-P − Thickness pre-PHA-P) − (Thickness post-PBS − Thickness pre-PBS).

This formula corrected for non-specific PBS-induced swelling, yielding a specific measure of the immune response to PHA-P. This approach provided a precise assessment of how dietary treatments influenced T-cell mediated immunity in broilers.

#### Antioxidant enzyme activities and blood cell count

Total antioxidant capacity (TAC) was measured using a Naxifer™ Total Antioxidant Capacity Assay Kit (Navand Salamat Co., Iran; Cat. No. NS-15012, NS-15013), based on the ferric reducing antioxidant power (FRAP) principle and single-electron transfer mechanism. The color change resulting from the reduction reaction was measured at 593 nm using a microplate reader (BioTek Instruments, USA).Catalase (CAT) activity was determined using a Nactaz™ Catalase Enzyme Activity Assay Kit (Navand Salamat Co., Iran), following the manufacturer’s instructions. The decomposition of hydrogen peroxide was monitored spectrophotometrically at approximately 520 nm.

Blood hepatic enzyme activities, including aspartate transaminase (AST) and alanine transaminase (ALT), were determined using an auto-analyzer spectrophotometer (BioSystems S.A., Barcelona, Spain). Approximately 2 mL of blood was collected in heparinized tubes, kept on ice, and used to prepare plasma and hemolysate for thiobarbituric acid reactive substances (TBARS) and glutathione peroxidase (GHS-Px) assays (Kei, 1978; Yagi, 1984). GHS-Px activity was measured using RANSEL and RANSOD kits (Randox Laboratories, United Kingdom). Plasma TBARS concentrations were expressed as nmol/mL.

At 42 days, 1 mL of whole blood was collected in heparinized capillary tubes for white blood cell (WBC) counts, including lymphocytes, monocytes, eosinophils, heterophils, and the heterophil-to-lymphocyte (H:L) ratio.

#### Blood lipid profile

Blood samples (2 mL) were collected from the wing vein of two birds per replicate on day 42 to obtain plasma. Plasma was analyzed for total cholesterol, triglycerides, high-density lipoprotein (HDL), low-density lipoprotein (LDL), and very low-density lipoprotein (VLDL) using diagnostic kits (Pars Azmon Co., Iran) with a spectrophotometer.

### Statistical analysis

All statistical analyses were performed using SAS software version 9.2 (SAS Institute Inc., Cary, NC, USA). The normality of data and homogeneity of variances were tested using the Shapiro–Wilk and Levene’s tests, respectively. When the assumptions of normality were not met, data were transformed prior to analysis. Percentage and proportional data (e.g., mortality rate) were subjected to arcsine square-root transformation, whereas antibody titers were expressed as log₂-transformed values before analysis to normalize the distribution of dilution-based data.

Continuous variables were analyzed using the Mixed Procedure (PROC MIXED), with dietary treatment as a fixed effect and pen as a random effect. For repeated measurements (blood metabolites, enzyme activities, CBC indices, and antibody titers at 28 and 42 days), candidate covariance structures—including compound symmetry (CS), first-order autoregressive (AR(1)), and unstructured (UN)—were compared, and the CS structure was selected based on the lowest Akaike information criterion (AIC). Antibody titers on day 0 were included as covariates to adjust for baseline differences. When transformed data were analyzed, least-squares means were back-transformed to the original scale for presentation. Duncan’s multiple range test was used for pairwise comparisons among treatment means.

Mortality data, expressed as the proportion of dead birds per pen, were analyzed using the GENMOD procedure to account for their binomial distribution. A logit link function was used, with dietary treatment as a fixed effect and pen as the experimental unit. Model fit was assessed using deviance and Pearson statistics. Treatment effects were determined based on P-values from the Wald chi-square test within the GENMOD framework, and least-squares means were back-transformed to percentages for presentation. Statistical significance was declared at *P* < 0.05, and 0.05 ≤ *P* < 0.10 was considered a trend.

## Results

### Nutritional composition of protein sources

The proximate composition and essential amino acid (EAA) profiles of BSFL, GH and SBM were assessed to evaluate their potential as feed ingredients (Table 1). BSFL and GH powders showed high crude protein (CP) levels at 61.47 % and 58.65 %, respectively, compared to 40.9 % in SBM, on a dry matter basis. Fiber content was 5.2 % in BSFL, 6.8 % in GH, and 3.1 % in SBM. Fiber content was 5.2 % in BSFL, 6.8 % in GH, and 3.1 % in SBM. EAA profiles (Table 2) confirmed that both BSFL and GH contain all essential amino acids needed for broiler growth, with GH particularly rich in lysine and methionine. Gamma irradiation did not significantly alter the proximate composition of BSFL/GH but improved microbial safety by reducing bacterial, mold, and yeast loads. These findings suggest that BSFL and GH powders can serve as viable alternatives to SBM in poultry diets, offering comparable or superior protein quality and amino acid profiles while also benefiting from enhanced microbial safety after irradiation

**Table 2:** Moisture content and essential amino acid composition of Soybean meal (SBM), gamma irradiated Black soldier fly larvae (BSFL) and Grasshopper (GH).

^1^According to AOAC (2016) protocols.	Feedstuff		
	BSFL	GH	SBM
	Moisture content, %		
	10	4.5	10.7
	Amino acid composition, % of dry matter		
Lysine	4.67	3.84	2.72
Methionine	1.96	2.32	0.63
Threonine	2.56	3.70	1.64
Valine	5.62	3.95	2.21
Isoleucine	4.20	3.91	3.40
leucine	5.47	4.36	5.10
Tyrosine	4.67	3.25	2.66
Phenylalanine	4.60	5.45	5.05
Histidine	2.43	3.14	2.50
Arginine	3.65	5.51	3.43
Tryptophan	0.84	0.79	0.71

Zeolite (natural clinoptilolite) was used as a multifunctional pelleting aid (pellet binder/flow agent and moisture adsorbent). Zeolite inclusion was adjusted slightly across treatments (absolute changes ≈ 0.3–0.8 percentage points depending on rearing phase and insect inclusion level) to maintain comparable pellet physical quality (cohesion, durability and flowability) after substitution of soybean meal with insect powders (Papaioannou et al., 2005, Suchý et al., 2006).

### Nutritional impact of gamma irradiation

Gamma irradiation effectively reduced total microbial counts in BSFL and GH powders (Table 3). At 0 kGy, total microbial counts were 1.02 × 10^2^ CFU/g for BSFL and 1.0 × 10 CFU/g for GH (means of three independent subsamples per treatment, *n* = 3). After exposure to 5 and 10 kGy, microbial counts in both powders fell below the method detection limit and are reported as <10 CFU/g. The method detection limit was 10 CFU/g and each subsample was plated in duplicate; values in Table 3 are means of independent replicates. The reduction in total microbial load following irradiation was statistically significant (*P* < 0.01)

**Table 3:** Total microbial count (CFU/g) of Black Soldier Fly Larvae (BSFL) and Grasshopper (GH) after exposure to different gamma ray irradiation dosages (kGy).

Gamma Irradiation (kGy)	Total microbial count (CFU/g)	
	BSFL	GH
0	10^2^	10
5	<10	<10
10	<10	<10

**Table 4:** Ingredients and chemical composition the experimental diets^1^.

Feed Ingredient, %	Starter							Grower						
	CON	BSF8	BSF16	BSF24	GH8	GH16	GH24	CON	BSF8	BSF16	BSF24	GH8	GH16	GH24
Corn	48.00	48.00	48.00	48.00	48.00	48.00	48.00	54.00	54.00	54.00	54.00	54.00	54.00	54.00
Corn Gluten Meal	4.00	3.15	2.37	1.47	3.27	2.59	1.87	4.32	4.00	3.31	2.60	3.98	3.40	3.00
Soybean Meal	32.00	29.44	26.88	24.32	29.44	26.88	24.32	24.20	22.26	20.33	17.96	22.26	20.04	17.96
Insect powder (BSF)	0.00	2.56	5.12	7.68	0.00	0.00	0.00	0.00	1.94	3.87	6.24	0.00	0.00	0.00
Insect powder (GH)	0.00	0.00	0.00	0.00	2.54	5.12	7.68	0.00	0.00	0.00	0.00	1.94	4.16	6.24
Wheat	4.50	5.00	5.00	7.00	5.00	5.50	7.00	5.68	6.95	7.41	7.60	6.90	7.28	7.50
Wheat bran	3.00	3.50	4.00	4.00	3.50	4.00	4.00	4.18	3.10	3.65	4.10	3.94	3.80	3.95
Vegetable Oil	2.20	1.95	1.90	1.25	1.85	1.50	0.90	1.65	1.40	1.25	1.20	1.21	1.10	0.95
Dicalcium phosphate	1.90	1.80	1.75	1.65	1.85	1.75	1.70	1.79	1.74	1.60	1.55	1.61	1.61	1.60
CaCO_3_	1.20	1.15	1.08	1.05	1.20	1.17	1.15	1.10	1.07	0.98	0.95	1.00	1.00	0.98
Common Salt	0.38	0.37	0.37	0.37	0.37	0.37	0.37	0.38	0.38	0.38	0.38	0.38	0.38	0.38
DL-Methionine	0.25	0.25	0.24	0.20	0.25	0.21	0.17	0.30	0.23	0.20	0.18	0.22	0.22	0.19
L-Lysine HCl	0.33	0.29	0.26	0.20	0.31	0.27	0.25	0.39	0.36	0.34	0.31	0.35	0.35	0.30
L-Threonine	0.11	0.10	0.10	0.10	0.08	0.05	0.00	0.16	0.13	0.13	0.12	0.08	0.10	0.07
Vitamin-Mineral Premix	0.50	0.50	0.50	0.50	0.50	0.50	0.50	0.50	0.50	0.50	0.50	0.50	0.50	0.50
Coccidiostat	0.05	0.05	0.05	0.05	0.05	0.05	0.05	0.05	0.05	0.05	0.05	0.05	0.05	0.05
Pellet binder	1.0	1.0	1.0	1.0	1.0	1.0	1.0	0.0	0.0	0.0	0.0	0.0	0.0	0.0
Zeolite	0.58	0.89	1.38	1.16	0.79	1.04	1.04	0.30	0.89	1.00	1.26	0.58	1.01	1.33
Celite	0.0	0.0	0.0	0.0	0.0	0.0	0.0	1.0	1.0	1.0	1.0	1.0	1.0	1.0
**Proximate Analysis**														
Metabolizable Energy (kcal/kg)^1^	2870	2870	2870	2870	2870	2870	2870	2950	2950	2950	2950	2950	2950	2950
Crude protein (%)	22.5	22.5	22.5	22.5	22.5	22.5	22.6	20.5	20.5	20.5	20.5	20.5	20.5	20.5
Ca (%)	1.0	1.0	1.0	1.0	1.0	1.0	1.0	0.9	0.9	0.9	0.9	0.9	0.9	0.9
P (%)	0.5	0.5	0.51	0.5	0.5	0.5	0.5	0.45	0.45	0.45	0.46	0.45	0.45	0.45
Lys (%)	1.2	1. 2	1.21	1.2	1.2	1.2	1.2	1.08	1.09	1.08	1.08	1.08	1.08	1.08
Met (%)	0.52	0.52	0.52	0.52	0.52	0.52	0.52	0.48	0.48	0.48	0.48	0.48	0.48	0.48
		
**Feed Ingredient, %**	**CON**	**BSF8**	**BSF16**	**BSF24**	**GH8**	**GH16**	**GH24**	**CON**	**BSF8**	**BSF16**	**BSF24**	**GH8**	**GH16**	**GH24**
Corn	55.00	55.00	57.00	57.00	55.50	56.00	57.00	57.00	57.00	58.00	57.80	57.10	57.00	57.80
Corn Gluten Meal	2.26	1.74	1.12	0.45	1.76	1.34	0.75	1.80	1.25	0.69	0.24	1.36	0.85	0.40
Soybean Meal	24.20	22.24	20.33	17.96	22.24	20.33	17.96	22.00	20.24	18.48	16.72	20.24	18.48	16.72
Insect powder (BSF)	0.00	1.96	3.87	6.24	0.00	0.00	0.00	0.00	1.76	3.52	5.28	0.00	0.00	0.00
Insect powder (GH)	0.00	0.00	0.00	0.00	1.96	3.87	6.24	0.00	0.00	0.00	0.00	1.76	3.52	5.28
Wheat	7.81	7.81	6.00	6.00	7.00	6.00	6.00	7.90	7.80	7.00	7.55	7.55	7.70	7.90
Wheat bran	2.20	2.20	3.20	3.80	3.10	3.80	3.80	2.71	3.36	4.00	4.00	3.30	4.10	4.00
Vegetable Oil	2.95	2.91	2.68	2.60	2.84	2.80	2.30	2.84	2.80	2.62	2.49	2.75	2.58	2.14
Dicalcium phosphate	1.53	1.47	1.40	1.33	1.45	1.40	1.34	1.50	1.44	1.40	1.36	1.45	1.40	1.34
CaCO_3_	0.97	0.93	0.91	0.85	0.98	0.96	0.96	1.00	0.97	0.92	0.89	0.99	0.98	0.98
Common Salt	0.38	0.38	0.38	0.36	0.36	0.36	0.36	0.38	0.38	0.36	0.36	0.36	0.36	0.36
DL-Methionine	0.21	0.20	0.18	0.16	0.18	0.16	0.13	0.21	0.20	0.18	0.17	0.19	0.16	0.14
L-Lysine HCl	0.31	0.31	0.23	0.18	0.28	0.26	0.23	0.31	0.27	0.23	0.19	0.29	0.26	0.24
L-Threonine	0.12	0.11	0.11	0.10	0.09	0.06	0.04	0.12	0.11	0.11	0.10	0.10	0.07	0.05
Vitamin-Mineral Premix	0.50	0.50	0.50	0.50	0.50	0.50	0.50	0.50	0.50	0.50	0.50	0.50	0.50	0.50
Coccidiostat	0.05	0.05	0.05	0.05	0.05	0.05	0.05	0.05	0.05	0.05	0.05	0.05	0.05	0.05
Pellet binder	1.0	1.0	1.0	1.0	1.0	1.0	1.0	1.0	1.0	1.0	1.0	1.0	1.0	1.0
Zeolite^4^	0.51	1.20	1.04	1.42	0.71	1.12	1.34	0.68	0.88	0.94	1.31	1.02	0.99	1.11
Celite	0.0	0.0	0.0	0.0	0.0	0.0	0.0	0.0	0.0	0.0	0.0	0.0	0.0	0.0
**Proximate Analysis**														
Metabolizable Energy (kcal/kg)^3^	3025	3025	3025	3025	3025	3025	3025	3025	3025	3025	3025	3025	3025	3025
Crude protein (%)	19	19	19	19	19	19	19	18.5	18.5	18.5	18.5	18.5	18.5	18.5
Ca (%)	0.78	0.78	0.78	0.78	0.78	0.78	0.78	0.78	0.78	0.78	0.78	0.78	0.78	0.78
P (%)	0.39	0.39	0.39	0.39	0.39.	0.39.	0.39.	0.39	0.39	0.39	0.39	0.39	0.39	0.39
Lys (%)	1.1	1.1	1.1	1.1	1.1	1.1	1.1	1.05	1.05	1.05	1.05	1.05	1.05	1.05
Met (%)	0.47	0.47	0.47	0.47	0.47	0.47	0.47	0.45	0.45	0.45	0.45	0.45	0.45	0.45

1 BSFL 8-24, GH 8-24: treatment groups in which the soybean meal was replaced with 8, 16 or 24 % of Black soldier fly larvae (BSFL) or grasshopper (GH), respectively. ^2^The following amounts were added to the diet per kg: 120 mg of Mn, 100 mg of Zn, 50 mg of Fe, 16 mg of Cu, 1.25 mg of I, 0.3 mg of Se, 10,000 IU of vitamin A, 5,000 IU of vitamin D_3_, 80 IU of vitamin E, 3.2 mg of menadione, 3.2 mg of thiamine, 8 mg of riboflavin, 65 mg of niacin, 20 mg of pantothenic acid, 4 mg of vitamin B_6_, 0.2 mg of biotin, 2.2 mg of folic acid, 0.02 mg of vitamin B_12_, and 1,700 mg of choline.

3 Metabolizable Energy (kcal/kg) values estimated using WUFFDA feed formulation software.

4 Zeolite (clinoptilolite) inclusion values shown represent manufacturing adjustments made solely to maintain pellet integrity after partial replacement of soybean meal with insect powders. These small modifications did not affect the calculated nutrient composition or the analyzed proximate values used for statistical comparisons.

### Production efficiency

Broilers fed diets containing BSFL and GH exhibited significant improvements in growth performance compared to the control group. The highest body weight was recorded in broilers fed the BSFL24 diet, particularly during the grower (15–25 d) and finisher (26–42 d) phases. European Production Efficiency Factor (EPEF) calculations (Table 5) indicated that broilers fed diets containing BSFL and GH powders demonstrated significant improvements across all rearing phases compared to the control group. During the starter phase (1–14 d), EPEF values were highest for GH16 and GH24 treatments, reflecting superior growth performance and feed efficiency. In the grower phase (15–25 d), BSFL24 recorded the greatest EPEF, followed by BSFL16 and GH16, all significantly outperforming the control (*p* < 0.05). The finisher I phase (26–35 d) showed a similar trend, with BSFL24, BSFL16, GH16, and GH24 treatments achieving significantly higher EPEF values, with BSFL24 emerging as the most efficient. This pattern continued into the finisher II phase (36–42 d), where BSFL24 maintained the highest EPEF, significantly surpassing all other dietary treatments. Over the entire 1–42 d period, total EPEF was highest for BSFL24, followed by BSFL16, GH16, and GH24, with all treatments significantly outperforming the control (*p* < 0.05). EPEF was calculated according to the formula described in the Materials and Methods section.These findings underscore the efficacy of BSFL24 as a dietary intervention for optimizing broiler performance.

**Table 5:** Mortality rate and European production efficiency factor of Arian broiler chickens fed with different dietary treatments.

Growth phase	Treatments^1^							SEM	*P*-Value
	CONL	BSF8	BSF16	BSF24	GH8	GH16	GH24		
	Mortality rate, %								
Starter (1-14 d)	0.0	0.0	0.0	0.0	0.0	0.0	0.0	0.0	>0.05
Grower (15-25 d)	0.0^b^	0.0^b^	0.0^b^	0.0^b^	1.6^a^	0.0^b^	0.0^b^	0.48	>0.05
Finisher I (26-35 d)	1.6	1.6	0	0	0	0	0	0.82	>0.05
Finisher II (36-42 d)	1.5	0	3.1	1.6	3.1	1.6	3.1	0.79	0.579
Entire period (1-42 d)	8.97^a^	2.56^b^	2.56^b^	2.56^b^	6.41^ab^	1.28^b^	2.56^b^	1.826	0.05
	**European production efficiency factor**								
Starter (1-14 d)	256.0^d^	266.0^c^	271.0^bc^	280.0^ab^	264.0^cd^	281.0^a^	281.0^a^	3.00	<0.0001
Grower (15-25 d)	363.5^d^	378.8^c^	390.0^bc^	404.7^a^	364.9^d^	381.2^c^	381.2^c^	4.17	<0.0001
Finisher I (26-35 d)	422.1^c^	438.0^bc^	458.9^a^	467.5^a^	437.3^c^	454.9^ab^	454.9^ab^	6.12	<0.0001
Finisher II (36-42 d)	343.8^b^	369.1^ab^	370.0^ab^	399.6^a^	332.2^b^	376.0^ab^	376.0^ab^	18.97	0.2644
Entire period (1-42 d)	345.6^c^	362.7^bc^	370.2^ab^	387.5^a^	342.1^c^	372.2^ab^	372.2^ab^	7.28	0.0008

1 BSFL8-16, GH8-16: Treatment groups in which soybean meal was replaced with 8 %, 16 %, or 24 % black soldier fly larvae (BSFL) or grasshopper (GH), respectively.

### Mortality rates and health

Mortality rates of broilers were assessed across rearing phases under the seven experimental treatments. During the starter (1–14 d) and finisher I (26–35 d) periods mortality did not differ among treatments (*P* > 0.05). In the grower period (15–25 d), mortality was reduced to 0 % in BSFL and GH treatments compared with 2.67 % in the control (*P* = 0.1135). In the finisher II period (36–42 d) the control showed higher mortality (3.95 %) than insect-fed groups (0–2.56 %), but this difference was not significant (*P* = 0.5797). Total mortality over 1–42 d was lower in BSFL and GH groups (1.28–6.41 %) than in the control (8.97 %), with the overall treatment effect approaching statistical significance (*P* = 0.0502; Table 5).

### Blood biochemical parameters

Serum enzyme activities, including AST and ALT, were unaffected by dietary treatments, indicating no adverse effects of BSFL or GH inclusion on liver function (*p* > 0.05; Table 6). Blood antioxidant enzyme activities (GHS-Px, CAT, and SOD) and thiobarbituric acid reactive substances (TBARS) levels showed no significant differences among treatments (Table 6). Importantly, the consistency in antioxidant enzyme activities suggests that dietary inclusion of BSFL and GH did not induce oxidative stress. This stability in oxidative balance highlights the potential of these insect-based protein sources to maintain broiler health without compromising the body's enzymatic defense mechanisms against oxidative damage. Blood lipid profiles were significantly influenced by dietary treatments (Table 7). The BSFL24 treatment resulted in the lowest cholesterol (120 mg/dL) and triglyceride levels (55 mg/dL), along with the highest HDL levels (60 mg/dL) and lowest LDL (40 mg/dL) and VLDL levels (11 mg/dL). These values were significantly better than those observed in the control and other treatment groups (p < 0.05).

**Table 6:** Blood antioxidant enzymes activities, hepatic enzyme profile and serum thiobarbituric acid reactive substances (TBARS) level in broilers fed different dietary treatments.

Items^2^	Treatments^1^								
	CONL	BSF8	BSF16	BSF24	GH8	GH16	GH24	SEM	*P*-Value
GHS-Px (U/mL)	295.20	298.80	307.20	329.00	288.60	296.40	314.40	18.10	0.74
CAT (U/mL)	90.01	89.74	96.18	100.94	92.61	97.27	95.22	5.19	0.72
SOD (U/mL)	188.4	189.3	198.6	210.6	188.8	196.1	200.0	9.88	0.19
TBARS (nmol/mL)	20.90	20.51	20.09	19.66	21.14	20.76	19.93	0.87	0.87
TAC (U/mL)	1564.60	1578.40	1617.20	1703.80	1570.20	1663.20	1667.00	81.01	0.81
AST (U/L)	209.60	205.20	198.60	202.60	213.80	197.20	193.80	8.78	0.99
ALT (U/L)	20.06	19.44	22.12	20.26	20.51	21.45	19.93	2.33	0.69

1 BSFL8-16, GH8-16: Treatment groups in which soybean meal was replaced with 8 %, 16 %, or 24 % black soldier fly larvae (BSFL) or grasshopper (GH), respectively.

2 SOD: Superoxide dismutase; CAT: catalase; GHS-Px: glutathione peroxidase; TBARS: thiobarbituric acid reactive substances; TAC: total antioxidant capacity; AST: aspartate transaminase; ALT: alanine transaminase.

**Table 7:** Effects of different dietary treatments on blood metabolites of broilers at the end of the experimental period (d 42).

Blood metabolites (mg/dl)	Treatments^1^							SEM	*P*-Value
	CONL	BSF8	BSF16	BSF24	GH8	GH16	GH24		
Cholesterol	152.8^a^	144.2^abcd^	140.4^bcd^	133.6^d^	152^ab^	145^abc^	139.4^cd^	4.07	0.025
TG	104.6^a^	89.1^bc^	76.3^cd^	73.7^d^	100^ab^	85.2^cd^	78^cd^	4.45	<0.0001
HDL	40.3^cd^	41.8^bcd^	45.1^abc^	46.7^a^	39.9^d^	44.4^abcd^	45.9^ab^	1.68	0.030
LDL	90.8^a^	84.3^ab^	80.5^b^	76.9^b^	86.5^ab^	84.4^ab^	81.6^ab^	3.52	0.180
VLDL	20.9^a^	17.8^bc^	15.3^cd^	14.7^d^	20^ab^	17^cd^	15.6^cd^	0.89	<0.0001

^1^BSFL8-16, GH8-16: Treatment groups in which soybean meal was replaced with 8 %, 16 %, or 24 % black soldier fly larvae (BSFL) or grasshopper (GH), respectively.

^a,b,c,d^ Different letters in a row indicate significant differences (*p* < 0.05).

### Immune responses

No significant differences (P > 0.05) were observed among dietary treatments in total or differential white blood cell counts at day 42 (Table 8). This indicates that dietary inclusion of BSFL or GH powders did not alter leukocyte profiles or immune balance, further supporting the safety and immunological stability of these insect-based protein sources in broiler diets. Serum antibody titers against AI, ND, and SRBC were significantly influenced by BSFL and GH supplementation (Table 9). At 28 days, broilers fed BSFL24 exhibited the highest AI and ND titers, significantly surpassing other treatments (*p* < 0.01). By 42 days, AI titers were highest in GH16 and GH24 treatments, while ND titers remained highest in BSFL24. SRBC titers were not significantly different at 28 days (*p* = 0.1230), but at 42 days, BSFL24 and GH8 treatments demonstrated significantly higher titers than the control (*p* < 0.0001). The wing web swelling index(WWSI), indicative of cellular immune responses, was highest in broilers fed BSFL16, GH24, and GH16, significantly exceeding the control group (*p* = 0.0711). Intermediate responses were observed in BSFL8 and BSFL24 treatments, suggesting a dose-dependent effect of BSFL supplementation on immune function. All antibody titers (AI, ND, and SRBC) are presented as log₂ (*n* = 12 birds per treatment; two birds per replicate). The serum antibody titers against AI, ND, SRBC, and the WWSI in response to PHA-P injection varied among dietary treatments. At 28 days, AI titers were significantly higher in BSF24 (5.1 log₂) compared to the control (3.8 log₂), with intermediate values in BSF16, GH8, GH16, and GH24 groups (*P* < 0.0001). By 42 days, AI titers peaked in GH16 and GH24 (5.3 and 5.1 log₂, respectively) and were significantly higher than in the control (4.6 log₂; *P* = 0.0007). ND titers followed a similar trend, with BSF24 showing the highest ND titer (5.8 log₂) at 28 days, while GH16 and GH24 exhibited comparable elevations. At 42 days, ND titers were highest in BSF24 (7.5 log₂), followed by grasshopper treatments GH16 and GH24 (6.2 log₂; *P* < 0.0001).

**Table 8:** Effect of different dietary treatments on white blood cells (WBCs) count at day 42.

	Treatments^1^								
WBCs count	CONL	BSF8	BSF16	BSF24	GH8	GH16	GH24	SEM	*P*-Value
WBCs, × 10^3^/mm^3^	15647	15765	14884	15696	15704	15220	16128	1669.978	0.9991
Lymphocytes, %	60.60	59.80	64.30	63.10	61.00	59.90	63.30	4.229	0.9787
Monocytes, %	4.40	4.80	4.40	4.20	4.60	4.40	4.80	0.529	0.9790
Eosinophils, %	0.6	0.4	0.4	0.4	0.6	0.6	0.4	0.245	0.9770
Heterophils, %	32.90	32.80	32.30	33.10	33.5	34.60	35.70	3.169	0.9894
H/L Ratio	0.54	0.55	0.50	0.52	0.55	0.58	0.58	0.061	0.9769

1 BSFL8-16, GH8-16: Treatment groups in which soybean meal was replaced with 8 %, 16 %, or 24 % black soldier fly larvae (BSFL) or grasshopper (GH), respectively.

**Table 9:** Serum titers against Avian Influenza (AI), Newcastle Disease (ND), Sheep Red Blood Cell (SRBC) antibodies and wing web swelling index (WWSI) in response to PHA injection.

Items	Treatments^1^							SEM	*P*-Value
	CON	BSF8	BSF16	BSF24	GH8	GH16	GH24		
AI titer (log _2_)									
28 d	3.8^c^	3.8^c^	4.5^b^	5.1^a^	4.7^ab^	4.4^b^	4.7^ab^	0.19	<0.0001
42 d	4.6^bc^	4.6^bc^	4.0^cd^	5.0^ab^	5.1^ab^	5.3^a^	5.1^ab^	0.24	0.0007
ND titer (log _2_)									
28 d	4.0^de^	4.2^cde^	4.6^c^	5.8^a^	5.2^b^	4.5^cd^	5.2^b^	0.18	<0.0001
42 d	5.2^c^	6.2^b^	5.3^c^	7.5^a^	6.2^b^	5.7^bc^	6.2^b^	0.21	<0.0001
SRBC titer (log _2_)									
28 d	3.2^ab^	2.8^b^	3.5^ab^	3.3^ab^	3.7^a^	2.9^b^	3.7^a^	0.24	0.1230
42 d	3.4^cd^	3.0^d^	4.3^b^	5.5^a^	5.0^a^	3.7^c^	5.0^a^	0.22	<0.0001
WWSI (mm)	2.75^c^	2.89^bc^	3.87^ab^	3.74^abc^	3.14^abc^	3.99^a^	4.03^a^	0.370	0.0711

1 BSFL8-16, GH8-16: Treatment groups in which soybean meal was replaced with 8 %, 16 %, or 24 % black soldier fly larvae (BSFL) or grasshopper (GH), respectively. ^a,b,c,d^ Different letters in a row indicate significant differences (*p* < 0.05).

SRBC titers, indicative of humoral immunity, showed no significant differences among treatments at 28 days (*P* = 0.1230). However, at 42 days, BSF24 and GH24 demonstrated significantly higher titers (5.5 and 5.0 log₂, respectively) than the control (3.4 log₂; *P* < 0.0001). The WWSI, a measure of cell-mediated immunity, did not differ statistically among groups (*P* = 0.0711). Nevertheless, numerical increases were observed, with the highest WWSI values recorded for GH16 (3.99 mm) and GH24 (4.03 mm), followed closely by BSF24 (3.74 mm).

### Antioxidant enzyme activities

Dietary treatments had no significant impact on blood antioxidant enzyme activities, including GHS-Px, CAT, and SOD (*p* > 0.05). Thiobarbituric acid reactive substances (TBARS) levels also remained unaffected, suggesting that dietary BSFL and GH did not compromise oxidative stability or induce oxidative stress. The unchanged levels of these antioxidant enzymes across treatments reinforce the notion that BSFL and GH inclusion do not exacerbate oxidative damage, a critical consideration in maintaining broiler health under varying dietary conditions.

### Cellular immune responses

The WWSI response to PHA-P injection varied significantly across treatments. The highest swelling indices were observed in broilers fed BSFL16, GH24, and GH16, while the lowest responses were recorded in the control group. BSFL24 and BSFL8 treatments produced intermediate responses, indicating dose-dependent effects of BSFL inclusion on cellular immunity. This enhanced immune response, coupled with the absence of oxidative stress markers, underscores the potential of BSFL and GH as sustainable and effective dietary interventions in broiler production.

## Discussion

### Nutritional impact of gamma irradiation

Known for their high-quality protein content and essential amino acids, BSFL have been shown to positively influence broiler health by improving gut microbiota composition, increasing short-chain fatty acid (SCFA) concentrations, and enhancing gut morphology (Bhattacharyya et al., 2024; Salahuddin et al., 2024). These improvements lead to better nutrient absorption and overall performance. The presence of bioactive compounds in BSFL—such as antimicrobial peptides, chitin, and lauric acid—further supports gut barrier function, limits pathogenic bacteria, and modulates immune responses (Vilela et al., 2023; Lau et al., 2024). While GH have received less research attention, they also offer valuable nutritional and functional properties. Their peptides and micronutrients may support gut health and immune function, contributing to improved feed efficiency and growth (Ndotono et al., 2022). The amino acid profiles of BSFL and GH, particularly their richness in lysine, methionine, and threonine, are crucial for protein synthesis, muscle growth, and immunity. Enhanced amino acid balance supports better protein utilization, leading to reduced FCR and increased production efficiency (Salahuddin et al., 2024). Gamma irradiation significantly reduced microbial contamination in both BSFL and GH powders. However, proximate composition and amino acid levels remained stable, confirming the process as a safe method for microbial control without compromising nutritional integrity.

### Production efficiency

This study demonstrated that incorporating BSFL and GH meals into broiler diets significantly enhanced production efficiency, as reflected in elevated EPEF scores. Notably, diets containing BSF24, GH16, and GH24 outperformed the control in terms of growth and feed utilization. These results are consistent with previous studies supporting the efficacy of insect-based proteins in poultry nutrition (Oonincx et al., 2015; Surendra et al., 2020; Loponte et al., 2017). The observed improvements in EPEF are attributed to the high-quality protein and essential amino acid content of BSFL and GH. Defatted BSFL, in particular, offers improved digestibility and energy utilization due to its reduced fat content (Makkar et al., 2014). Replacing 16 % or 24 % of SBM with BSFL or GH enhanced performance outcomes, supporting their use as sustainable alternatives to conventional protein sources (Bovera et al., 2015; Makkar et al., 2014). All experimental diets were isocaloric and isonitrogenous, isolating the impact of protein source. BSFL’s digestible proteins and bioactive compounds positively influenced gut morphology, enzyme activity, and microbial balance, all of which are critical for nutrient absorption and growth (Cutrignelli et al., 2018; Bhattacharyya et al., 2024; Elahi et al., 2022). Additionally, functional components such as chitin, antimicrobial peptides, and polyunsaturated fatty acids enhanced immune function and suppressed gut pathogens (Salahuddin et al., 2024; Chobanova et al., 2024).

GH also improved feed efficiency at both 16 % and 24 % inclusion levels. Though less studied than BSFL, GH is a source of valuable bioactive lipids and peptides that support gut integrity and immune health (Lee et al., 2024).

The amino acid composition of BSFL and GH further reinforces their utility. BSFL is notably rich in lysine, methionine, and threonine, which are vital for protein synthesis and muscle development (Dörper et al., 2024). Methionine also supports DNA methylation, an essential metabolic function. GH contributes high levels of arginine and valine, which are important for muscle repair and protein synthesis (Vilela et al., 2023).

While gamma irradiation did not alter nutrient composition, it indirectly supported performance by improving microbial safety. This may contribute to better gut health and nutrient utilization in broilers. Overall, incorporating BSFL and GH at 16–24 % as isonitrogenous and isocaloric feed components optimized production efficiency. The BSFL24 group achieved the highest EPEF, confirming BSFL’s potential as a high-performance feed ingredient. GH also demonstrated effectiveness, particularly at higher inclusion levels, reinforcing its value as a sustainable protein source. These outcomes align with global efforts to enhance poultry productivity while reducing reliance on traditional feed ingredients (Lau et al., 2024). This study evaluated BSFL and GH meal inclusion up to 24 % of soybean meal replacement. Testing higher inclusion levels beyond the present experiment levels are sugessted in future research to determine the upper physiological and economic limit for insect-based proteins use in broiler diets*.*

### Mortality rates and health

The integration of insect-based meals into broiler diets during early growth stages has generally shown no adverse effects on survival rates. Studies indicate that BSFL at inclusion levels up to 15 % did not significantly impact mortality, feed intake, or body weight gain during the starter phase, though performance tended to improve numerically with higher inclusion (Triandoyo et al., 2022). Similarly, *Tenebrio molitor* and silkworm pupae meals had no detrimental effects on survival, supporting their safety and efficacy as alternative protein sources (Sedgh-Gooya et al., 2022; Zsedely et al., 2023).

In the present study, the overall broiler mortality was not significantly affected across treatments. However, a clear reduction in mortality during the grower phase was noted in BSFL and GH groups, particularly in BSF8 and GH16, likely due to the immunomodulatory and antimicrobial effects of bioactive compounds such as chitin and antimicrobial peptides. These components may enhance gut health and disease resistance, especially under commercial rearing conditions.

During the finisher phase, slight mortality increases in BSF16, GH8, and GH24 treatments may reflect potential challenges related to palatability or nutrient balance, indicating the need for optimization at higher inclusion levels. Nonetheless, the notably low total mortality in BSF8 and GH16 groups suggests these inclusion levels strike an effective balance between nutrition and gut health benefits. Furthermore, insect meals have been linked to improved gut microbiota composition, notably increasing beneficial bacteria like *Lactobacillus* and *Roseburia*, which are associated with enhanced immune function and nutrient absorption (Vilela et al., 2023; Ndotono et al., 2022). Although overall mortality did not differ significantly, a slight increase in mortality was observed during the finisher II period (control 3.95 % vs insect-fed groups 0–2.56 %; *P* = 0.2644). This mortality “bump” may stem from late-phase thermal/environmental stress (older, heavier birds are more sensitive to ambient fluctuations) (Wasti et al., 2020), metabolic or cardiovascular burdens associated with rapid growth. Given that insect meals were gamma-irradiated and microbial contamination was minimized, feedborne pathogens are unlikely the cause. Future trials should include postmortem examination and pathogen screening to pinpoint causes more precisely.

### Blood biochemical parameters

Serum biochemical markers offer valuable insights into broiler health, particularly liver function and lipid metabolism (Lumeij, 2008; Nunes et al., 2018). In the present study, the inclusion of gamma-irradiated BSFL and GH led to notable improvements in lipid profiles without adversely affecting liver enzymes.

Diets enriched with BSFL—especially at the 24 % inclusion level—significantly reduced serum cholesterol (133.6 mg/dL) and triglycerides (73.7 mg/dL), while increasing HDL levels (46.7 mg/dL). GH meal inclusion also improved lipid profiles, though less markedly. These beneficial shifts in lipid parameters may be mediated by multiple factors. Insect meals contain chitin and chitosan derivatives that have been shown *in vitro* to bind bile acids and lipids, and in some animal studies chitosan/chitin supplementation reduced serum cholesterol and triglycerides (Khoushab and Yamabhai, 2010, Razdan, 1994, Zhou et al., 2020). In addition, microbial fermentation of insect-derived substrates can increase short-chain fatty acids and modulate lipid metabolism, and insect lipids (notably medium-chain fatty acids such as lauric acid) may directly influence hepatic lipid handling (Borrelli et al., 2017; Cullere et al., 2016). Evidence from poultry is promising but not yet conclusive; therefore we present these mechanisms as plausible contributors rather than proven causal pathways in broilers. Such modulation contributes to enhanced lipid metabolism and overall metabolic health. Liver health, assessed via AST and ALT enzyme levels, remained unaffected by the inclusion of BSFL or GH, corroborating findings from Sypniewski et al. (2020) in turkeys and Gasco et al. (2018) in rabbits. This indicates that these insect-based diets do not induce hepatic stress or injury. Benzertiha et al. (2019) also reported reduced liver triglycerides and mass in broilers fed insect oils compared to those given palm or poultry fats, linking the effect to the higher PUFA content of insect-derived fats. Since PUFAs suppress lipogenesis in the liver (Ferramosca et al., 2012), their presence in BSFL and GH likely contributes to the observed benefits

The triglyceride-lowering effects observed in this study align with previous reports showing reduced serum TG levels when saturated fats are replaced with PUFAs in poultry diets (Viveros et al., 2009; Wongsuthavas et al., 2007). Given that all diets were iso-caloric and iso-nitrogenous, the metabolic improvements can be confidently attributed to the insect meal inclusion rather than other dietary variations.

Collectively, these findings affirm the potential of BSFL and GH—especially in their gamma-irradiated forms—as functional feed ingredients that support lipid regulation, liver health, and metabolic stability, further highlighting their suitability for sustainable broiler production

### Immune responses

Incorporating BSFL and GH into broiler diets significantly enhanced humoral immunity, as reflected by elevated antibody titers against AI, ND, and SRBC. Among all treatments, BSF24 and GH16/GH24 yielded the strongest effects, with BSF24 producing the highest AI and ND titers at both 28 and 42 days. This enhancement is likely due to bioactive compounds—such as antimicrobial peptides, chitin, and fatty acids—that promote B-cell activation, antibody synthesis, and antigen presentation (Biasato et al., 2020; De Marco et al., 2015). Chitin and its derivative chitosan, in particular, have well-documented immunomodulatory effects, including the stimulation of T-helper cells.

GH diets, especially GH16 and GH24, also led to increased AI titers, suggesting that GH possesses a comparable bioactive profile to BSFL and can serve as a viable alternative protein source. SRBC titers at day 28 were not significantly affected by the dietary treatments (*P* = 0.1230. However, by day 42, broilers fed BSFL and GH diets—particularly BSF24 and GH8—showed significantly higher SRBC titers than the control group (*P* < 0.0001). This delayed response suggests that prolonged exposure to insect-derived bioactive components, including chitin and antimicrobial peptides, may enhance B-cell activation and secondary antibody production over time (Biasato et al., 2020; De Marco et al., 2015). These results are consistent with prior studies demonstrating the immune-enhancing properties of insect-based diets across several species, including poultry (Lee et al., 2018b), turkeys (Sypniewski et al., 2020), pigs (Spranghers et al., 2018; Wang and Shelomi, 2017), and fish (Weththasinghe et al., 2021). Bioactive elements such as antimicrobial peptides, phenoloxidase, and lysozyme in BSFL appear to support both humoral and cellular immune mechanisms (Salahuddin et al., 2024; Bruno et al., 2021). High antibody titers in BSF24 birds—5.1 log₂ for AI at 28 days and 7.5 log₂ for ND at 42 days—underscore the immunostimulatory potential of BSFL. In terms of oxidative status, no significant changes were observed in antioxidant enzyme activities (CAT, GHS-Px, TBARS, TAC) or liver enzyme markers (AST, ALT), indicating that BSFL and GH supplementation did not induce oxidative stress or hepatic strain. These findings align with earlier reports showing that insect-based diets can deliver high-quality protein and functional compounds like chitin and peptides without disturbing oxidative homeostasis (Biasato et al., 2020; De Marco et al., 2015).

Contrastingly, Chen et al. (2022) found adverse effects on liver and kidney function with BSFL inclusion levels exceeding 75 %. The discrepancy may stem from excessive inclusion rates or differing rearing conditions. In the current study, moderate levels of BSFL and GH did not alter GHS-Px, TBARS, or SOD levels, suggesting preserved antioxidant defenses.

Chitin and chitosan are known to exhibit antioxidative properties. Chitosan oligosaccharides (COS), for example, have been shown to enhance CAT and SOD activities while reducing lipid peroxidation in broilers under thermal stress (Lan et al., 2020; Lan et al., 2024; Fathi et al., 2023). Other studies highlight chitosan’s hepatoprotective effects, including its ability to safeguard liver enzymes from oxidative injury (Rezaei Koochacksaraei et al., 2020; Özdek et al., 2022; Login et al., 2009). No significant changes were found in antioxidant markers of healthy broilers, suggesting that BSFL and GH diets maintain oxidative balance and liver function. The presence of chitin and chitosan may offer added protection, especially under stress. Their known antioxidative and hepatoprotective effects support their potential to enhance resilience in birds facing environmental or physiological challenges. Further research is needed to confirm their long-term benefits under such conditions.

### Cellular immune responses

The inclusion of defatted BSFL and GH in iso-caloric and iso-nitrogenous broiler diets did not significantly affect hematological parameters, including WBC count, lymphocyte, monocyte, eosinophil, heterophil percentages, or the heterophil-to-lymphocyte (H/L) ratio. This stability indicates that these insect-based protein sources did not induce physiological stress or immune disturbances, suggesting their suitability as alternative feed ingredients.

The maintenance of blood cell profiles across treatments may be attributed to bioactive components in BSFL and GH, such as chitin, chitosan, and antimicrobial peptides, which contribute to immune homeostasis (De Marco et al., 2015; Biasato et al., 2020). The unchanged H/L ratio, a reliable stress marker in poultry, further supports that the diets were well-tolerated and non-stressful (De Marco et al., 2013). Previous studies confirm that defatted BSFL supports gut and immune health without negative impacts on immunity or stress parameters (Secci et al., 2018), while GH meal also provides nutritional benefits without compromising immune function (Schiavone et al., 2018).

Although WWSI, a proxy for T-cell-mediated immunity, did not differ significantly, numerical improvements in GH16, GH24, and BSF24 suggest enhanced cellular immune response. Chitosan has been shown to stimulate cytokine production and promote T-cell proliferation (Bovera et al., 2016), aligning with these trends.

PHA-induced swelling responses were significantly enhanced in diets containing 16 % and 24 % BSFL or GH, suggesting a dose-dependent effect of chitin. Chitin-derived chito-oligosaccharides may activate pattern recognition receptors such as TLRs, leading to T-cell activation and proliferation (Lee et al., 2018b; Wolf and Underhill, 2018). The prebiotic effects of chitin may further support systemic immunity by modulating gut microbiota, fostering beneficial bacterial populations (van Huis, 2020; Makkar et al., 2014).

Variability in PHA response, particularly at higher inclusion levels, may reflect individual physiological differences. However, the consistent trend of immune enhancement across insect-fed groups supports the robustness of these effects. Compared to soybean meal-based control diets, BSFL and GH inclusion supported T-cell function and provided immunological benefits alongside nutritional value.

Additionally, BSFL has shown immunostimulatory effects in other species. For instance, it enhanced macrophage phagocytic activity and antibody titers against avian influenza in quails (Harlystiarini et al., 2020) and increased CD4+ *T* lymphocyte counts and lysozyme activity (Lee et al., 2018b). However, mixed results have been reported for humoral immunity against specific poultry diseases such as ND and infectious bursal disease (Park et al., 2023), indicating that immune modulation may be parameter-specific.

Nevertheless, the ability of BSFL to replace conventional protein sources without impairing growth or immunity supports its utility in sustainable poultry nutrition (Attia et al., 2023). Importantly, both BSFL and GH inclusion significantly improved production efficiency, especially at higher inclusion levels (BSF24 and GH24), as evidenced by enhanced EPEF values. These improvements, particularly during the starter phase, likely contributed to sustained growth performance and underline the functional potential of insect meals as both nutritional and immunological enhancers.

## Conclusion

The findings of this study indicate that incorporating gamma-irradiated BSFL and GH powder into broiler diets can enhance growth performance, immune function, and lipid metabolism without negatively affecting oxidative balance. Birds receiving 24 % irradiated insect meals achieved the highest production efficiency and reduced mortality, highlighting the nutritional and functional value of these alternative protein sources. Gamma irradiation likely contributed to improved nutrient digestibility and microbial safety, enhancing the bioavailability of key compounds such as chitin and chitosan. These bioactives supported humoral and cellular immune responses while maintaining antioxidant enzyme stability. Collectively, the results support the use of irradiated insect meals as safe and effective alternatives to soybean meal in broiler diets, with clear benefits for growth performance, immunity, and nutrient utilization.

## CRediT authorship contribution statement

**Ali Reyan Mohassesi:** Writing – review & editing, Writing – original draft, Formal analysis. **Hassan Darmani Kuhi:** Writing – review & editing, Validation, Supervision, Resources, Project administration, Methodology, Investigation, Funding acquisition, Formal analysis, Data curation, Conceptualization. **Ardeshir Mohit:** Writing – review & editing. **Shahrokh Ghovvati:** Writing – review & editing, Validation, Software, Methodology, Data curation.

## Disclosures

The authors declare that they have no known competing financial interests or personal relationships that could have appeared to influence the work reported in this paper.
